# Understanding of Depressive Symptomatology across Major Depressive Disorder and Bipolar Disorder: A Network Analysis

**DOI:** 10.3390/medicina60010032

**Published:** 2023-12-24

**Authors:** Hyukjun Lee, Junwoo Jang, Hyo Shin Kang, Jakyung Lee, Daseul Lee, Hyeona Yu, Tae Hyon Ha, Jungkyu Park, Woojae Myung

**Affiliations:** 1 Department of Neuropsychiatry, Seoul National University Bundang Hospital, Seongnam 13620, Republic of Korea; lhj4071@gmail.com (H.L.); julian6312@gmail.com (J.J.); jklee96@ewhain.net (J.L.); dasulee627@gmail.com (D.L.); hkhkh9274@gmail.com (H.Y.); hatti@snu.ac.kr (T.H.H.); 2 Department of Psychology, Kyungpook National University, Daegu 41566, Republic of Korea; hyoshin.kang@knu.ac.kr; 3 Department of Psychiatry, Seoul National University College of Medicine, Seoul 03087, Republic of Korea

**Keywords:** bipolar disorder, data analysis, depression, major depressive disorder, mood disorders

## Abstract

*Background and Objectives:* Depressive symptoms are prominent in both major depressive disorder (MDD) and bipolar disorder (BD). However, comparative research on the network structure of depressive symptoms in these two diagnostic groups has been limited. This study aims to compare the network structure of depressive symptoms in MDD and BD, providing a deeper understanding of the depressive symptomatology of each disorder. *Materials and Methods:* The Zung Self-Rating Depressive Scale, a 20-item questionnaire, was administered to assess the depressive symptoms in individuals with MDD (*n* = 322) and BD (*n* = 516). A network analysis was conducted using exploratory graph analysis (EGA), and the network structure was analyzed using regularized partial correlation models. To validate the dimensionality of the Zung SDS, principal component analysis (PCA) was adopted. Centrality measures of the depressive symptoms within each group were assessed, followed by a network comparison test between the two groups. *Results:* In both diagnostic groups, the network analysis revealed four distinct categories, aligning closely with the PCA results. “Depressed affect” emerged as the most central symptom in both MDD and BD. Furthermore, non-core symptoms, “Personal devaluation” in MDD and “Confusion” in BD, displayed strong centrality. The network comparison test did not reveal significant differences in the network structure between MDD and BD. *Conclusions:* The absence of significant differences in the network structures between MDD and BD suggests that the underlying mechanisms of depressive symptoms may be similar across these disorders. The identified central symptoms, including “Depressed affect”, in both disorders and the distinct non-core symptoms in each highlight the complexity of the depressive symptomatology. Future research should focus on validating these symptoms as therapeutic targets and incorporate various methodologies, including non-metric dimension reduction techniques or canonical analysis.

## 1. Introduction

Depressive symptoms are common psychiatric features of mood disorders, including major depressive disorder (MDD) and bipolar disorder (BD). MDD and BD—with a lifetime prevalence of 16% and 5%, respectively—are common conditions associated with reduced quality of life, increased mortality, and risk of suicide [1,2,3,4,5,6,7]. Both MDD and BD are debilitating conditions, with frequent recurrence observed in approximately 85% and over 90% of individuals, respectively, and imposing significant personal and societal burdens [8,9,10].

While MDD and BD share the diagnostic criterion of depressive episodes within the Diagnostic and Statistical Manual of Mental Disorders, Fifth Edition (DSM-5) framework, they exhibit distinct differences [11]. To address the diagnostic ambiguity, the DSM-5 introduced a transdiagnostic mixed-features specifier, emphasizing a continuum, and alternative classifications such as “research-based diagnostic criteria” for a mixed major depressive episode (MDE) have also emerged [12,13]. In the clinical course, BD typically exhibits earlier onset, more frequent and shorter duration of depressive episodes, and greater short-term mood variability compared to MDD [14]. Symptomatically, depressive episodes in MDD are characterized by features such as decreased appetite, early insomnia, somatization, anxiety, agitation, and anger, whereas those in BD often exhibit irritability, psychotic symptoms, diurnal variation, and atypical symptoms [14,15,16,17,18,19]. Despite these distinctions, distinguishing between MDD and BD remains a challenge for mental health professionals due to the absence of clear differences. The fact that only 20% of patients with BD receive an accurate diagnosis when they experience depressive episodes, with 60% initially being diagnosed with MDD, highlights the difficulty in differentiating between these disorders [20,21]. Moreover, such misdiagnoses result in poorer outcomes, increased suicide risk, manic episodes, and increased healthcare costs [21,22,23,24,25,26].

Network analysis, based on a causal system perspective, prioritizes the possibility that the co-occurrence of symptoms arises from interactions between symptoms rather than a shared underlying cause, which sets it apart from traditional psychopathology [27,28]. Network analysis allows for exploring how the symptoms of mental disorders influence each other within a network framework [29]. This approach, known as network psychiatry, utilizes computational methods. It proves particularly valuable for analyzing heterogeneous conditions such as mood disorders—as it helps evaluate the contributions of individual symptoms to adaptive functioning within the network—and assessing distinctive, interconnected, or potential acting symptoms as risk factors for chronicity [30,31,32].

To date, network analyses comparing MDD and BD remains limited, despite their clinical importance [33]. Previous studies have had mixed findings. One has highlighted anergia and psychomotor speed as a central feature of BD compared to a group including MDD [33]. Meanwhile, another study did not reveal significant differences in depressive symptoms between the groups [34]. In the present study, we aimed to compare the network structures of self-rating depression severity scales in patients with MDD and BD to elucidate the central depressive symptoms in both groups and explore the connections among the clustering of symptoms.

## 2. Materials and Methods

### 2.1. Sample

A total of 838 participants (MDD [*n* = 322], BD [*n* = 516]) were included in the study. Individuals with a raw score <40 on the Zung Self-Rating Depression Scale (SDS) were excluded, as this study primarily focused on depressive symptoms in MDD and BD. All the diagnoses were made by board-certified psychiatrists (W.M. and T.H.H.) based on structured diagnostic interviews using the Mini-International Neuropsychiatric Interview (M.I.N.I) and through a review of case records or other available data [35]. Additionally, the diagnoses were established following the criteria outlined in the DSM-5 [11]. Demographic data and information related to the depressive symptom scales were collected from the patients.

### 2.2. Measurement

#### The Zung Self-Rating Depression Scale

The Zung SDS is a 20-item self-report questionnaire in which 10 items are positively worded and the other 10 are scored in reverse [36]. Responses are rated on a scale from 1 to 4, and the raw score (ranging from 20 to 80) is transformed into an index score between 25 and 100. Zung’s index score categorizes depression severity as follows: 50–59 for mild-to-moderate depression, 60–69 for moderate-to-severe depression, and ≥70 for severe depression [37]. The Zung SDS was designed to assess a wide range of depressive symptoms, including both unipolar and bipolar depression, and it is used to evaluate depressive symptoms not only in MDD but also in BD [38,39].

### 2.3. Statistical Analysis

#### 2.3.1. Network Construction and Community Detection

In the network, nodes represent items from the Zung SDS, while edges represent the bivariate partial correlation coefficients between items. These coefficients, expressed as edge weights, reflect the degree of connection between two nodes considering the influence of all the other nodes [40]. Nodes with stronger connections are positioned closer together, and thicker edges indicate a higher level of relevance. Within the network structure, nodes with stronger and more connections tend to be located at the center, while nodes with weaker and fewer connections are often found at the periphery. An undirected network was calculated and visualized using a Gaussian Graphical Model, which is suitable for a cross-sectional study design [41].

In order to build a reliable and parsimonious network, the Graphical Least Absolute Shrinkage and Selection Operator (glasso) method was employed. This technique effectively eliminates spurious edges and retains only robust associations by shrinking smaller edge weights to zero, thereby controlling for false positive edges and regularizing the network. The analysis utilized the EBICglasso function from the R-package “qgraph” [42,43].

Exploratory Graph Analysis (EGA) was used to identify distinct item communities within the network structure [44,45]. EGA is a data-driven community detection method that explores the underlying dimensionality of the items, without relying on prior knowledge. In comparison to traditional analytic methods such as factor analysis, EGA represents a more advanced and visually intuitive way to identify dimensions and reveal intricate associations within multivariable data [46]. In order to identify the optimal community structure for the Zung SDS items, the Louvain algorithm was employed, optimizing the hierarchically structured modularity measure between vertices within these item-based communities [47] For the evaluation of the item stability and the dimensionality of the network, a parametric bootstrapping procedure was executed. A total of 1000 bootstrap samples were generated from a multivariate normal distribution, ensuring the results from the EGA reflected the original dataset [45]. The EGA and bootEGA functions from the EGAnet package were utilized to perform these procedures [48]. To validate the dimensionality of the Zung SDS, traditional data reduction method—principal component analysis (PCA)—was adopted, and its solution was compared with the community structure for the Zung SDS obtained from the EGA.

#### 2.3.2. Centrality Estimation

To identify and evaluate the centrality of key symptoms in patients with MDD and BD, several centrality indices were employed: (1) node strength centrality, which measures the overall importance of specific symptoms in the network and is known as reliability; (2) node closeness centrality, indicating how well a symptom indirectly connects with others; and (3) node betweenness centrality, which highlights how frequently a symptom acts as a bridge between other symptoms following the shortest paths [49].

#### 2.3.3. Network Stability and Robustness

The network stability and the robustness of the centrality were evaluated using the correlation stability coefficient. This coefficient indicates the maximum proportion of cases that can be removed while maintaining a correlation of 0.7 with the original data, with a 95% probability [43]. A network is considered stable if most samples can be removed without a significant decrease in the centrality indices, with values between 0 and 1. Values >0.25 indicate moderate stability, while values >0.50 indicate strong stability [43]. Bootstrapped difference tests were conducted to evaluate the differences in both the strength centrality and edge weights between all the possible pairs of nodes using 1000 bootstrapping samples. Significant differences in the centrality indices and edge weights were determined via the 95% bootstrap CIs. Specifically, narrow bootstrapping CIs suggest that the estimates are precise, and if the CI contains a zero, it indicates that there is no significant difference in the centrality indices and edge weights between the two nodes. These procedures were carried out using the R-package “botnet” [50].

#### 2.3.4. Network Comparisons between MDD and BD

To compare the network structures between the MDD and BD groups, network comparison tests (NCTs) were conducted. These tests assessed the differences in the network structure (i.e., network invariance), the number of edges with significant differences (i.e., edge invariance), and overall network connectivity (i.e., global strength) between the two groups. Subsequently, Holm–Bonferroni correction was applied to account for multiple comparisons [51].

## 3. Results

### 3.1. Demographic and Clinical Characteristics

Table 1 shows the demographic data of the patients with MDD and BD enrolled in this study. The cohort had a mean age of 34.39 years old (SD = 12.55) and was predominantly female (72.2%) (Supplementary Figure S1). Based on the Bonferroni correction, we identified significant differences in age, marital status, and alcohol use status between the MDD and BD groups. Specifically, individuals with BD were younger, less frequently married, and more often engaged in alcohol use compared with their counterparts with MDD. Table 2 quantifies the responses to the Zung SDS across both diagnostic categories (Supplementary Figure S2). No significant mean differences were found in the SDS item scores between the MDD and BD groups after being subjected to Bonferroni correction for multiple comparisons.

### 3.2. Network and Community Estimation

**Figure 1:**
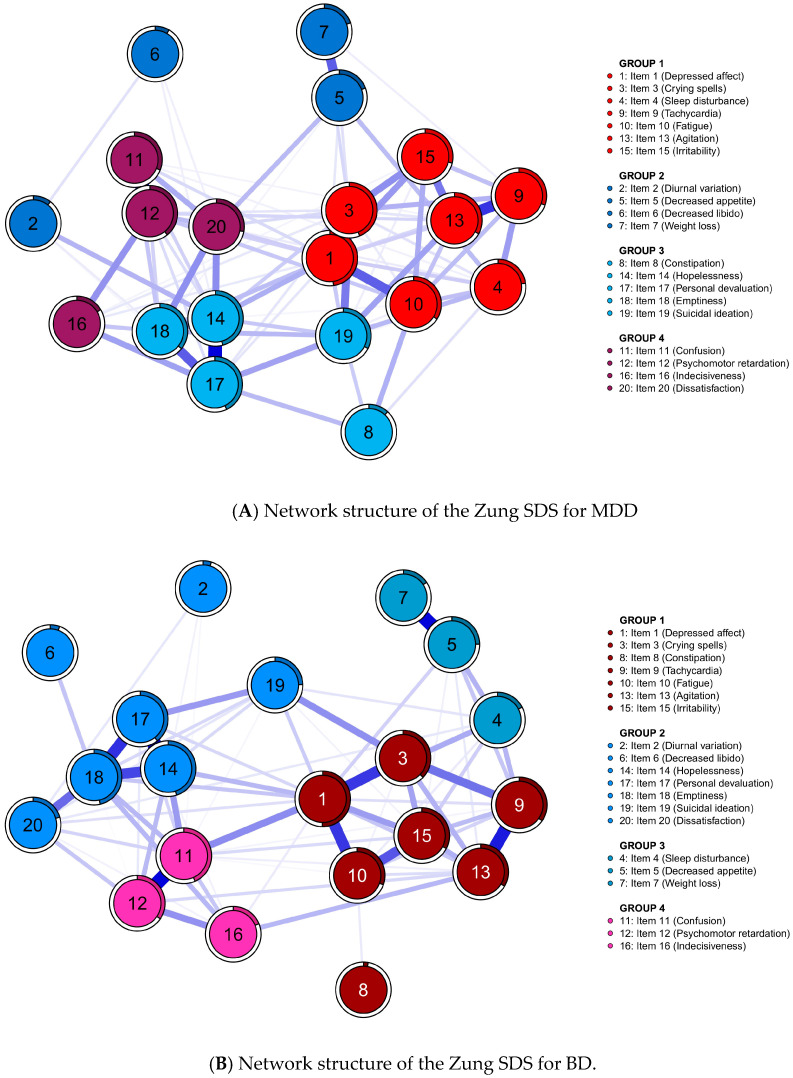
Network structure of the Zung SDS 20-symptom items for (**A**) MDD (*n* = 322) and (**B**) BD (*n* = 516). Each node (1 to 20) belongs to one of four distinct cluster. Description of their items’ symptoms are presented on the right side of the network. The thickness and saturation of the edges represent weights indicate the strength of the connection between two nodes. BD, bipolar disorder; MDD, major depressive disorder; SDS, Self-Rating Depression Scale.

Figure 1 illustrates the estimated symptom networks for both the MDD and BD groups. As shown in Figure 1, four distinct communities were discerned within each diagnostic group. In the MDD cohort, Community 1 comprised items 1, 3, 4, 9, 10, 13, and 15; Community 2, items 2, 5, 6, and 7; Community 3, items 8, 14, 17, 18, and 19; and Community 4, items 11, 12, 16, and 20. On the other hand, the BD cohort’s Community 1 included items 1, 3, 8, 9, 10, 13, and 15; Community 2, items 2, 6, 14, 17, 18, 19, and 20; Community 3, items 4, 5, and 7; and Community 4, items 11, 12, and 16. The results of the bootstrapped iterations showed that the derived four-dimension solution exhibits a replication rate of 74.7% (747 out of 1,000) in MDD and 74.8% (748 out of 1000) in BD.

### 3.3. Principal Component Analysis

To confirm the robustness of the item dimensions identified in the EGA, the EGA results were compared with those of the PCA. In the PCA analysis, four dimensions were identified, as shown in the scree plots (Supplementary Figures S3 and S4), which was an identical solution to the EGA. The patterns of the component loadings revealed that most items loaded on the same components as grouped in the EGA, confirming the dimensional robustness across the two methods, except for a few items, including item 11, 12, 16 and 19 in the BD group and item 4, 6, 8 and 20 in the MDD group. In particular, the patterns of the component loadings on PC1 and PC2 are similar in both the MDD and BD groups, while PC3 in MDD and PC4 in BD are distinct from each other, thus they might be considered as potential bases for discriminating between BD and MDD (Supplementary Tables S1 and S2). Supplementary Figures S5 and S6 display the visual representation of the network nodes, with PC1 and PC2 as the X-Y axes for the BD and MDD groups. These results demonstrate a high degree of similarity between the item clusters identified for the groups with distinct disorders by both the EGA and PCA, while some individual items were differently grouped between the identified clusters.

### 3.4. Centrality Indices and Edge Weights

**Figure 2:**
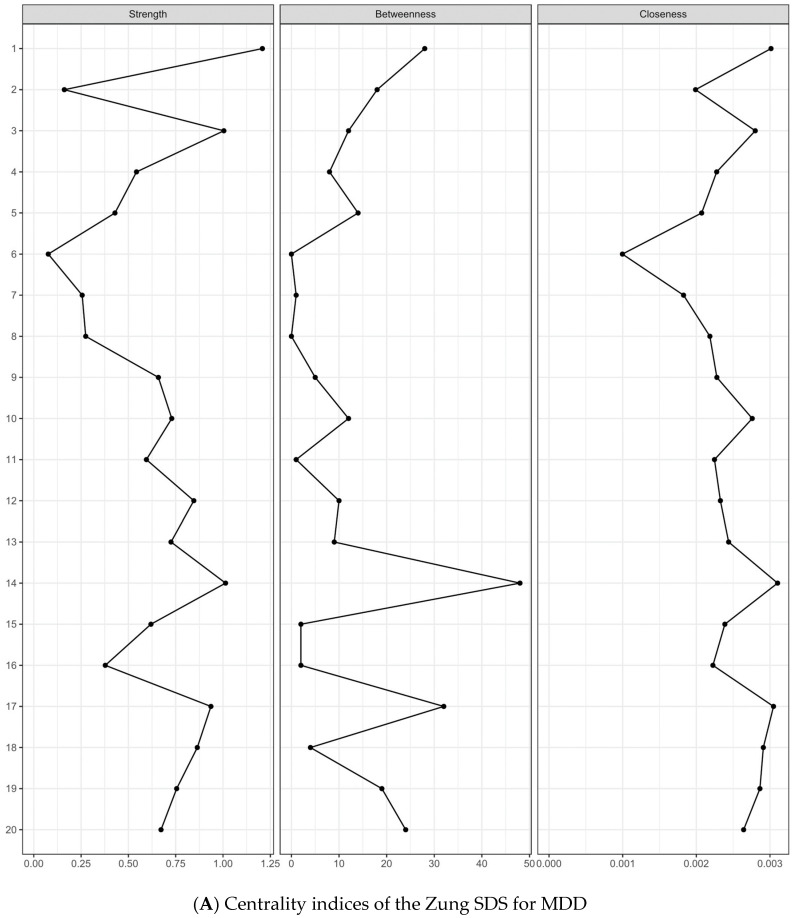

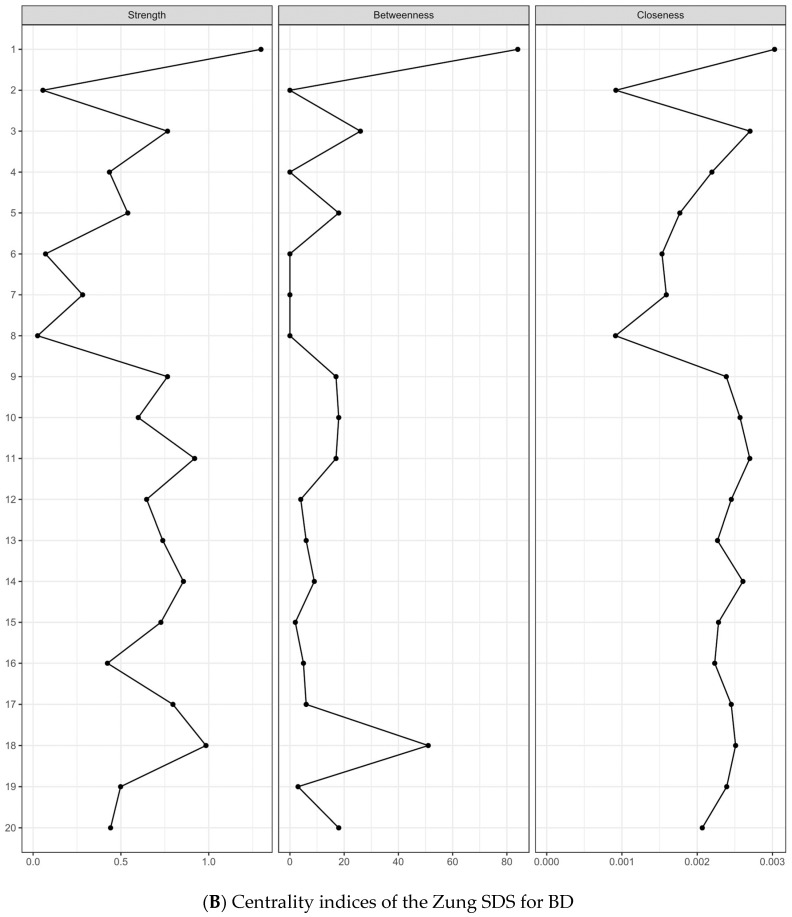
Centrality indices of the Zung SDS 20-symptom-items network for (**A**) MDD and (**B**) BD. The x-axis represents the standardized z-scores, and the y-axis represents the Zung SDS 20-symptom items. BD, bipolar disorder; MDD, major depressive disorder; SDS, Self-Rating Depression Scale.

The centrality indices for the study are outlined in Figure 2, highlighting that Item 1 (“Depressed affect”) consistently exhibited- high centrality across all the metrics in both the MDD and BD groups. Specifically, in the MDD group, the metrics were strength = 1.208, betweenness = 56, and closeness = 0.0030, whereas in the BD group, they were strength = 1.297, betweenness = 168, and closeness = 0.0030. In the MDD group, Items 14 (“Hopelessness”), 3 (“Crying spell”), 17 (“Personal devaluation”), and 18 (“Emptiness”) followed in the centrality metrics. In the BD group, Items 18 (“Emptiness”), 11 (“Confusion”), 14 (“Hopelessness”), 3 (“Crying spell”), and 9 (“Tachycardia”) followed. Notably, the bootstrapped difference tests confirmed the relative importance of these items (Supplementary Figures S7 and S8). In the MDD group, no significant differences in the strength centrality were observed among Items 1, 14, 3, 17, and 18 (Supplementary Figure S7). However, in the BD group, significant differences were found between Item 1 and Items 18, 11, 14, 3, and 9 (Supplementary Figure S8). Additionally, the bootstrapped difference tests for the edge weight (Supplementary Figures S9 and S10) showed that the edges between Item 11 (“Confusion”) and Item 12 (“Psychomotor retardation”), and between Item 14 (“Hopelessness”) and Item 17 (“Personal devaluation”), were most robust in the MDD group. Similarly, the edges between Item 14 (“Hopelessness”) and Item 17 (“Personal devaluation”), and between Item 11 (“Confusion”) and Item 12 (“Psychomotor retardation”), were most prominent in the BD group.

### 3.5. Network stability

In our investigation into the network stability, we assessed the indices of strength centrality in both patient groups—those with MDD and those with BD (Supplementary Figures S11 and S12). Our findings revealed that the correlation coefficient of 0.7 was maintained until 28.3% and 59.5% of the sample were removed in the MDD and BD groups, respectively. These results suggest a moderate-to-excellent level of strength stability in both groups.

### 3.6. Network Comparisons

In the comparative network analysis between patients with MDD and those with BD, we observed non-significant differences across the three pivotal metrics. Specifically, the overall network structure was not significantly different between the MDD and BD patients (M = 0.14, *p* = 0.623), while the difference in the global strength was also not significant as well (S = 0.309, *p* = 0.775). Additionally, no edges were found to be significant in the edge comparison tests.

## 4. Discussion

This study presents a network analysis using the Zung SDS, a severity rating scale for depressive symptoms, to explore the differences between MDD and BD. Our results revealed no significant differences in the results of the symptom severity scales or NCT within the network analysis, consistent with a previous study comparing MDD and BD [34]. This suggests that solely distinguishing between MDD and BD based on the characteristics of depressive episodes is a significant challenge.

In both the MDD and BD groups, Item 1 (“Depressed affect”) exhibited the strongest centrality in the network analysis. This finding aligns with previous network analyses of depression and the DSM-V, wherein “Depressed affect” is classified as a core symptom [11,52,53,54]. Moreover, “Depressed affect” not only has the highest impact on psychosocial functioning impairment among depressive symptoms but also serves as the most potent predictor of a depression diagnosis [55,56]. These observations are consistent with its centrality in our study.

We found that certain symptoms exhibited strong centrality despite not being included in the DSM-5 as core symptoms. Specifically, the symptoms “Personal devaluation” in MDD and “Confusion” in BD were identified as having strong centrality. “Personal devaluation” has well-established consistency in MDD [57,58]. Furthermore, a systematic review of network analyses of depressive symptoms in MDD revealed that the symptom of “Worthlessness” held significant centrality, in accordance with the results of the present study [59]. In BD, “Confusion” corresponds to the “Diminished ability to think or concentrate” in the DSM-5 and it is the second most commonly reported symptom among patients with mood disorder, following mood-related symptoms [60]. Cognitive symptoms in patients with BD are associated with a more severe prior course of illness, a higher number of manic episodes, more frequent hospitalizations, and a longer overall illness duration [61]. Despite not conforming to the DSM core symptom criteria, the strong centrality of these symptoms suggests that they should be carefully considered when assessing the interconnections and chronicity of depressive symptoms in patients with MDD and those with BD.

The symptoms that exhibited strong centrality in both groups, specifically “Emptiness” (Item 18) and “Hopelessness” (Item 14), indeed reflect the core symptoms of the DSM-5 (Supplementary Figures S7 and S8). “Hopelessness” is a symptom that encompasses both state and trait aspects [62]. It has long been a major focus of research on the etiology of depressive disorders related to cognitive vulnerabilities [62]. Longitudinal studies have shown that the symptom of “Hopelessness” plays a mediating role in the relationship between low levels of social support and increases in depressive symptoms, indicating its role not only as a symptom but also as a causal factor in MDD. Additionally, it is understood that “Emptiness” is a symptom serving as a defense mechanism to prevent psychotic regression and profound suffering, closely associated with social and occupational functional impairments [63,64,65,66]. The specificity of these symptoms of “Hopelessness” and “Emptiness” may have influenced the findings of this study.

In the NCT analysis, there were no significant differences between the MDD and BD groups. As indicated by previous findings, it is challenging to differentiate between these two groups based solely on the patterns of depressive symptoms [34,67]. This difficulty is reinforced by the absence of significant results in terms of the symptom mean differences in our study. Consequently, distinguishing between MDD and BD within an MDE necessitates a comprehensive, longitudinal evaluation, extending beyond a mere cross-sectional evaluation of depressive symptoms. This evaluation should consider associated factors, including comorbid anxiety and psychotic features, family history, substance use, medication response, seasonality, and history of suicide, with a recognition that variables such as alcohol use and family history may contribute to the differentiation, as revealed in our results [20,67,68,69,70].

Regarding the clustering and edge connections, Items 1, 3, 14, and 18 (“Depressed affect”, “Crying spell”, “Hopelessness” and “Emptiness”) align with the first criteria of the DSM-5 MDE symptoms, and the present study consistently demonstrates strong centrality for all four of these items. However, among the four symptoms, only “Hopelessness” and “Emptiness” are separately clustered in Group 2, aligning with prior research showing a strong association between these two symptoms (Figure 1) [71]. In addition, “Personal devaluation” also exhibits a robust edge with “Hopelessness” and “Emptiness” in both the MDD and BD groups. The relationships between “Hopelessness” and “Personal devaluation” are in line with Beck’s cognitive triad concept [72]. Consequently, “Emptiness”, despite being categorized as the first criterion of the DSM-5 MDE symptoms, presents distinct dimensions compared with the affective valence of “Depressed affect” and “Crying spell”. Furthermore, Items 11, 12, and 16 (“Confusion”, “Psychomotor retardation”, and “Indecisiveness”) are symptoms that cluster in both the MDD and BD groups, a finding that is in line with previous research on cognitive factors, demonstrating interconnectedness [73]. Moreover, the assessment of “Suicidal ideation” as a psychiatric emergency symptom reveals strong connections with “Personal devaluation”. Prior research has also established that “Personal devaluation” is a predictor of suicidal ideation and attempts, underscoring its significance in treatment [74,75,76].

This study must be interpreted in light of several limitations. First, network analysis is an evolving approach with enormous potential [29,77]. However, there are concerns about model selection, as well as uncertainties regarding reliability and replicability [78,79,80]. Particularly, in the analysis of symptom networks, selecting which symptoms to include is crucial; nevertheless, there is currently no consensus on which symptomatology to employ [33]. It is expected that ongoing research on various symptom clusters will lead to a consensus in this regard. Furthermore, to enhance the reliability and robustness of the study results, future investigations should consider extending the analysis by using non-metric dimension reduction techniques and canonical analysis. This extension is anticipated to contribute to elucidating the central depressive symptoms in both groups and exploring the connections among the clustering of symptoms. Secondly, due to the cross-sectional design of this study, the network analysis remains undirected, revealing associations but not establishing causal inferences. Consequently, it remains inconclusive whether the central symptom caused other adjacent depressive symptoms or vice versa. Additionally, due to the relatively small sample size, challenges in interpreting the closeness and betweenness compared to the strength of items persist. The potential for bias is also present due to the predominance of females in the total sample and the significant demographic differences between MDD and BD, such as age and alcohol use status. While previous studies have indicated no sex-based differences in the network structure, future research with a larger and more diverse sample is necessary for a more detailed analysis of these variables [81]. Thirdly, although there were general similarities, some differences in individual items were observed between the EGA and PCA results in this study. Future research should concentrate on integrating and comparing those two methods, emphasizing their dimensionality and loadings. Such an integrated approach would provide valuable insights into the underlying structures of depressive symptoms in MDD and BD.

## 5. Conclusions

This study aimed to explore the distinction in depressive symptoms between MDD and BD and to gain a deeper understanding of depressive symptomatology in both disorders using the depressive severity self-rating scale and network analysis. The results did not reveal significant differences in the depressive symptom severity between the two groups. In both MDD and BD, “Depressed affect” emerged as the most influential node. Additionally, certain symptoms that do not align with the core symptoms of an MDE per the DSM-5 exhibited strong centrality, that is, “Personal devaluation” in the MDD group and “Confusion” in the BD group. The present study demonstrated the challenge of differentiating between MDD and BD solely based on depressive symptoms. However, by identifying the influential symptoms and their associations, the study has contributed to an enhanced understanding of the depressive symptomatology within MDD and BD. Future research should focus on a diverse symptomatology range and consider adopting various models, including non-metric dimension reduction techniques, as well as canonical analysis with a larger sample size and a longitudinal evaluation of symptoms to better observe changes in symptomatology. Additionally, while the primary focus of this study was on the centrality in the network analysis, the results not only highlighted the similarities but also revealed some differences between PCA and EGA. This suggests the need for further research integrating these two analytical methods. Furthermore, the centrality findings from this study suggest the need for effectiveness validation of evidence-based interventions targeting symptoms with strong centrality.

## Figures and Tables

**Table 1:** Clinical and demographic characteristics of participants (*n* = 838).

Characteristics	Total Sample Mean ± SD, *n* or %	MDD Mean ± SD, *n* or %	BD Mean ± SD, *n* or %	Test Statistics	Bonferroni Corrected *p*-Value
Age (years)	34.39 ± 12.55	38.21 ± 13.36	32.00 ± 11.38	7.18	<0.001 ***
Gender (%)				0.90	1.00
Male	27.8	29.8	26.6		
Female	72.2	70.2	73.4		
Education (%)				2.97	0.68
High school or below	31.7	35.4	29.5		
Others	68.3	64.6	70.5		
Employment status (%)				1.39	1.00
Unemployed	45.1	47.8	43.4		
Employed	54.9	52.2	56.6		
Marital status (%)				12.10	<0.05 *
Married	30.8	37.3	26.7		
Others (Single, divorced, or widowed)	69.2	62.7	73.3		
Alcohol use status (%)				11.93	<0.01 **
Former or current	55.5	47.8	60.3		
Never	44.5	52.2	39.7		
Smoking status (%)				0.94	1.00
Past or current	32.6	47.8	33.9		
Never	67.4	52.2	66.1		
Psychiatric familial history	45.6	35.1	52.1	22.52	<0.001 ***

Note. * *p* < 0.05, ** *p* < 0.001, *** *p* < 0.001. Abbreviation: BD, bipolar disorder; MDD, major depressive disorder; SDS, Self-Rating Depression Scale.

**Table 2:** Analysis of item scores of Zung SDS between major depressive disorder and bipolar disorder.

	Items	Mean (SD)		U_Statistic	Bonferroni Corrected
		MDD (*n* = 322)	BD (*n* = 516)		*p*-Value
1	I feel down-hearted and blue.	2.79 (0.93)	2.78 (0.81)	83,384	1.00
2	Morning is when I feel the best.*	3.58 (0.74)	3.46 (0.82)	89,542.	0.49
3	I have crying spells or feel like it.	2.31 (0.90)	2.26 (0.86)	84,531	1.00
4	I have trouble sleeping at night.	2.58 (1.05)	2.54 (1.06)	84,691	1.00
5	I eat as much as I used to.*	2.50 (1.06)	2.38 (1.06)	88,546	1.00
6	I still enjoy sex.*	3.68 (0.66)	3.58 (0.72)	90,013	0.20
7	I notice that I am losing weight.	1.53 (0.79)	1.45 (0.77)	88,408	1.00
8	I have trouble with constipation.	1.65 (0.91)	1.76 (1.0)	78,888	1.00
9	My heart beats faster than usual.	2.25 (0.88)	2.29 (0.91)	80,663	1.00
10	I get tired for no reason.	2.90 (0.90)	2.99 (0.93)	78,298	1.00
11	My mind is as clear as it used to be.*	3.40 (0.82)	3.51 (0.78)	76,939	0.75
12	I find it easy to do the things I used to.*	3.33 (0.86)	3.48 (0.77)	75,421	0.22
13	I am restless and can’t keep still.	2.02 (0.87)	2.13 (0.89)	77,251	1.00
14	I feel hopeful about the future.*	3.36 (0.81)	3.41 (0.74)	81,242	1.00
15	I am more irritable than usual.	2.45 (0.96)	2.40 (0.96)	85,791	1.00
16	I find it easy to make decisions.*	3.11 (0.92)	3.23 (0.86)	77,804	1.00
17	I feel that I am useful and needed.*	3.17 (0.88)	3.26 (0.85)	78,033	1.00
18	My life is pretty full.*	3.55 (0.71)	3.60 (0.65)	80,991	1.00
19	I feel that others would be better off if I were dead.	1.62 (0.90)	1.77 (0.99)	76,624	0.74
20	I still enjoy the things I used to do.*	3.29 (0.84)	3.41 (0.72)	78,220	1.00
	Total score	55.08 (8.69)	55.67 (7.86)	79,783	1.00

* Inversely coded items. Abbreviation: BD, bipolar disorder; MDD, major depressive disorder; SDS, Self-Rating Depression Scale.

## Data Availability

The data presented in this study are available on request from the corresponding author (wmyung@snu.ac.kr). The data are not publicly available due to containing patients’ medical information.

## Supplementary Materials

The following supporting information can be downloaded at: https://www.mdpi.com/article/10.3390/medicina60010032/s1, Figure S1. Raincloud plot of ages between patients with major depressive disorder and bipolar disorder. Figure S2. Raincloud plot of total Zung SDS score between patients with major depressive disorder and bipolar disorder. Figure S3. Scree plot illustrating principal component analysis for patients with major depressive disorder. Figure S4. Scree plot illustrating principal component analysis for patients with bipolar disorder. Figure S5. Network plot of Zung SDS from principal component analysis loadings in patients with major depressive disorder. Figure S6. Network plot of Zung SDS from principal component analysis loadings in patients with bipolar disorder. Figure S7. Bootstrapped difference tests between nodes in the Zung SDS 20-symptom-items network among major depressive disorder patients. Figure S8. Bootstrapped difference tests between nodes in the Zung SDS 20-symptom-items network among bipolar disorder patients. Figure S9. Bootstrapped difference tests between edge-weights that were in the Zung SDS 20-symptom-items network among major depressive disorder patients. Figure S10. Bootstrapped difference tests between edge-weights that were in the Zung SDS 20-symptom-items network among bipolar disorder patients. Figure S11. Bootstrapped the strength centrality stability of the Zung SDS 20-symptom-items network among major depressive disorder patients. Figure S12. Bootstrapped the strength centrality stability of the Zung SDS 20-symptom-items network among bipolar disorder patients. Table S1. Component loadings of Zung Self-Rating Depression Scale in major depressive disorder. Table S2. Component loadings of Zung Self-Rating Depression Scale in bipolar disorder.
