# Supplementary material for: Understanding of Depressive Symptomatology across Major Depressive Disorder and Bipolar Disorder: A Network Analysis
Source: Medicina (Kaunas). 2023 Dec 24;60(1):32. doi: 10.3390/medicina60010032 (PMC10818784; doi:10.3390/medicina60010032)
Supplement: Supplementary file 1 [file medicina-60-00032-s001.zip › medicina-2766824-supplementary.pdf]

Supplementary Materials

Supplementary Table

Supplementary Table 1. Component loadings of Zung Self-Rating Depression Scale in major depressive disorder.

Supplementary Table 2. Component loadings of Zung Self-Rating Depression Scale in bipolar disorder.

Supplementary Figures

Figure S1. Raincloud plot of ages between patients with major depressive disorder and bipolar disorder.

Figure S2. Raincloud plot of total Zung SDS score between patients with major depressive disorder and bipolar disorder.

Figure S3. Scree plot illustrating principal component analysis for patients with major depressive disorder.

Figure S4. Scree plot illustrating principal component analysis for patients with bipolar disorder.

Figure S5. Network plot of Zung SDS from principal component analysis loadings in patients with major depressive disorder.

Figure S6. Network plot of Zung SDS from principal component analysis loadings in patients with bipolar disorder.

Figure S7. Bootstrapped difference tests between nodes in the Zung SDS 20-symptom-items network among major depressive disorder patients.

Figure S8. Bootstrapped difference tests between nodes in the Zung SDS 20-symptom-items network among bipolar disorder patients.

Figure S9. Bootstrapped difference tests between edge-weights that were in the Zung SDS 20-symptom-items network among major depressive disorder patients.

Figure S10. Bootstrapped difference tests between edge-weights that were in the Zung SDS 20-symptom-items network among bipolar disorder patients.

Figure S11. Bootstrapped the strength centrality stability of the Zung SDS 20-symptom-items network among major depressive disorder patients

Figure S12. Bootstrapped the strength centrality stability of the Zung SDS 20-symptom-items network among bipolar disorder patients.

Table S1. Component loadings of Zung Self-Rating Depression Scale in major depressive disorder.

	Items	PC1	PC2	PC3	PC4
**1**	I feel down-hearted and blue.	**0.590**	0.268	0.085	0.052
**2**	Morning is when I feel the best.	-0.199	0.047	**0.361**	0.026
**3**	I have crying spells or feel like it.	**0.712**	0.137	-0.210	0.004
**4**	I have trouble sleeping at night.	**0.440**	-0.089	0.064	0.223
**5**	I eat as much as I used to.	0.082	-0.064	0.170	**0.791**
**6**	I still enjoy sex.	-0.374	0.042	**0.539**	0.266
**7**	I notice that I am losing weight.	0.036	-0.010	-0.115	**0.751**
**8**	I have trouble with constipation.	0.153	-0.311	**0.361**	0.052
**9**	My heart beats faster than usual.	**0.726**	-0.191	0.039	0.112
**10**	I get tired for no reason.	**0.670**	-0.057	0.105	-0.172
**11**	My mind is as clear as it used to be.	0.282	0.254	**0.451**	-0.065
**12**	I find it easy to do the things I used to.	0.127	0.170	**0.547**	-0.058
**13**	I am restless and can’t keep still.	**0.654**	-0.116	0.113	0.051
**14**	I feel hopeful about the future.	-0.026	**0.760**	0.177	-0.078
**15**	I am more irritable than usual.	**0.713**	0.001	-0.060	0.019
**16**	I find it easy to make decisions.	0.200	0.022	**0.490**	-0.223
**17**	I feel that I am useful and needed.	-0.070	**0.793**	0.098	-0.065
**18**	My life is pretty full.	-0.031	**0.685**	0.287	0.031
**19**	I feel that others would be better off if I were dead.	0.207	**0.534**	-0.193	0.201
**20**	I still enjoy the things I used to do.	0.020	0.211	**0.474**	0.128
	Eigen value	3.41	2.47	1.95	1.50

Note. * Bold values indicate the highest component loading among all components.Abbreviation: PC, principal component

Table S2. Component loadings of Zung Self-Rating Depression Scale in bipolar disorder.

	Items	PC1	PC2	PC3	PC4
**1**	I feel down-hearted and blue.	**0.563**	0.172	0.127	0.079
**2**	Morning is when I feel the best.	0.000	0.136	-0.266	**0.676**
**3**	I have crying spells or feel like it.	**0.564**	0.151	0.160	-0.088
**4**	I have trouble sleeping at night.	**0.412**	0.127	0.083	-0.036
**5**	I eat as much as I used to.	0.028	-0.047	**0.659**	0.001
**6**	I still enjoy sex.	0.116	-0.132	-0.167	**0.735**
**7**	I notice that I am losing weight.	0.064	-0.129	**0.857**	-0.503
**8**	I have trouble with constipation.	0.227	**0.511**	-0.385	-0.338
**9**	My heart beats faster than usual.	**0.741**	-0.223	0.158	-0.086
**10**	I get tired for no reason.	**0.748**	-0.028	-0.245	0.230
**11**	My mind is as clear as it used to be.	0.066	0.229	0.245	**0.307**
**12**	I find it easy to do the things I used to.	0.082	0.263	**0.311**	0.260
**13**	I am restless and can't keep still.	**0.741**	-0.108	0.040	-0.007
**14**	I feel hopeful about the future.	-0.037	**0.770**	-0.099	0.177
**15**	I am more irritable than usual.	**0.829**	-0.273	0.010	0.079
**16**	I find it easy to make decisions.	-0.029	**0.401**	0.306	-0.182
**17**	I feel that I am useful and needed.	-0.128	**0.926**	-0.121	-0.047
**18**	My life is pretty full.	-0.170	**0.813**	0.091	-0.045
**19**	I feel that others would be better off if I were dead.	**0.422**	0.396	-0.110	-0.087
**20**	I still enjoy the things I used to do.	0.012	**0.354**	0.270	0.227
	Eigen value	3.37	3.02	1.70	1.56

Note. * Bold values indicate the highest component loading among all components. Abbreviation: PC, principal component.

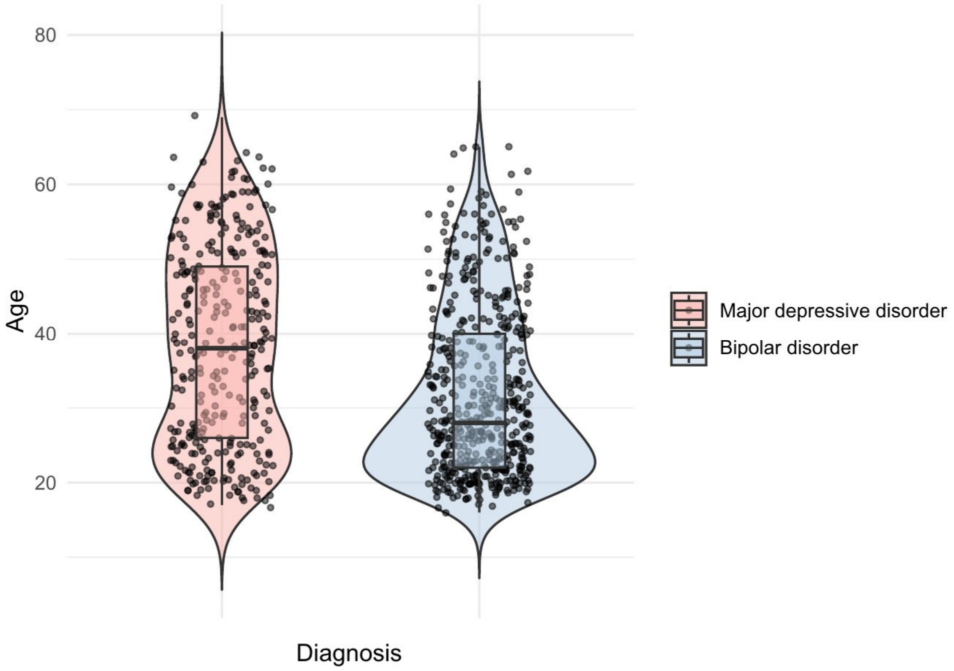

Figure S1. Raincloud plot of ages between patients with major depressive disorder and bipolar disorder.

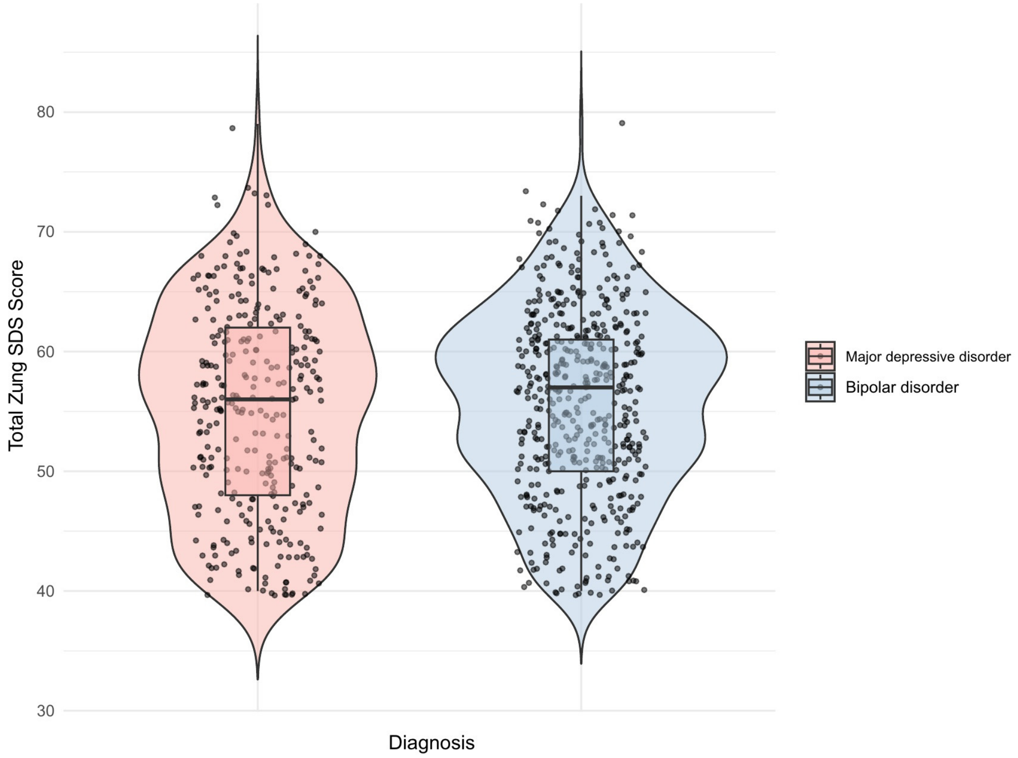

Figure S2. Raincloud plot of total Zung SDS score between patients with major depressive disorder and bipolar disorder. Abbreviation: SDS, Self-Rating Depression Scale

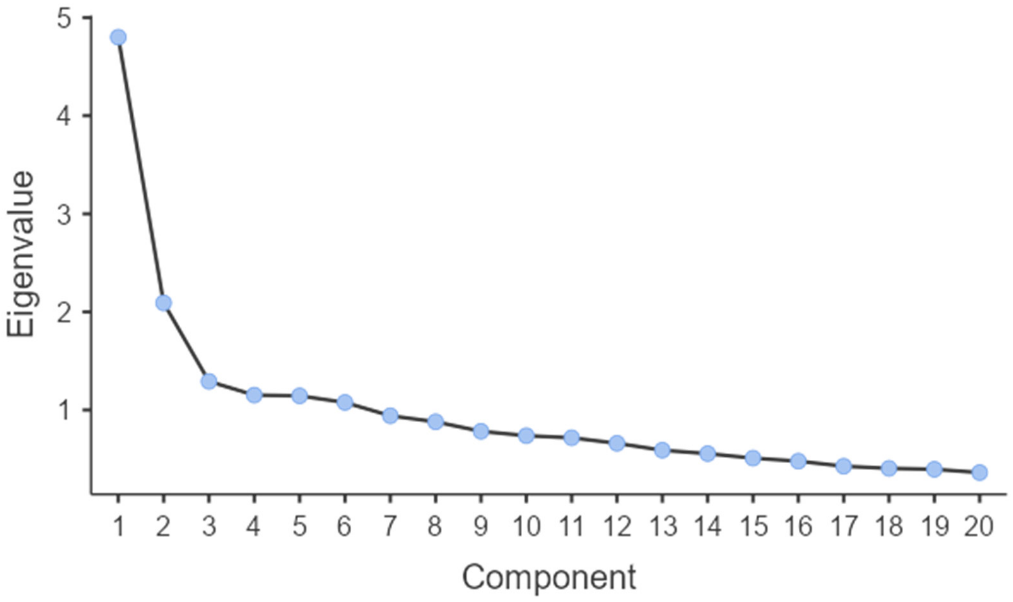

Figure S3. Scree plot illustrating principal component analysis for patients with major depressive disorder.

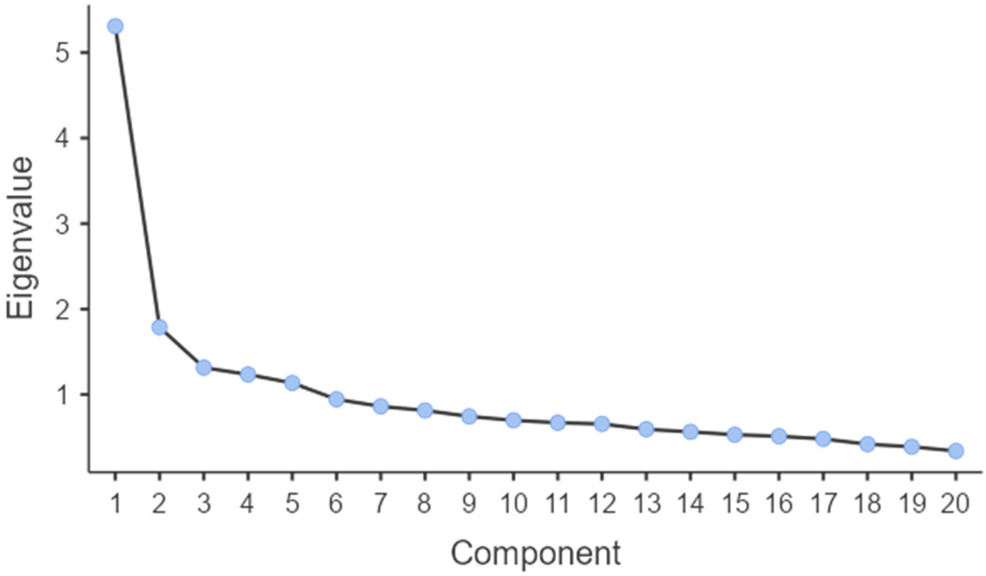

Figure S4. Scree plot illustrating principal component analysis for patients with bipolar disorder.

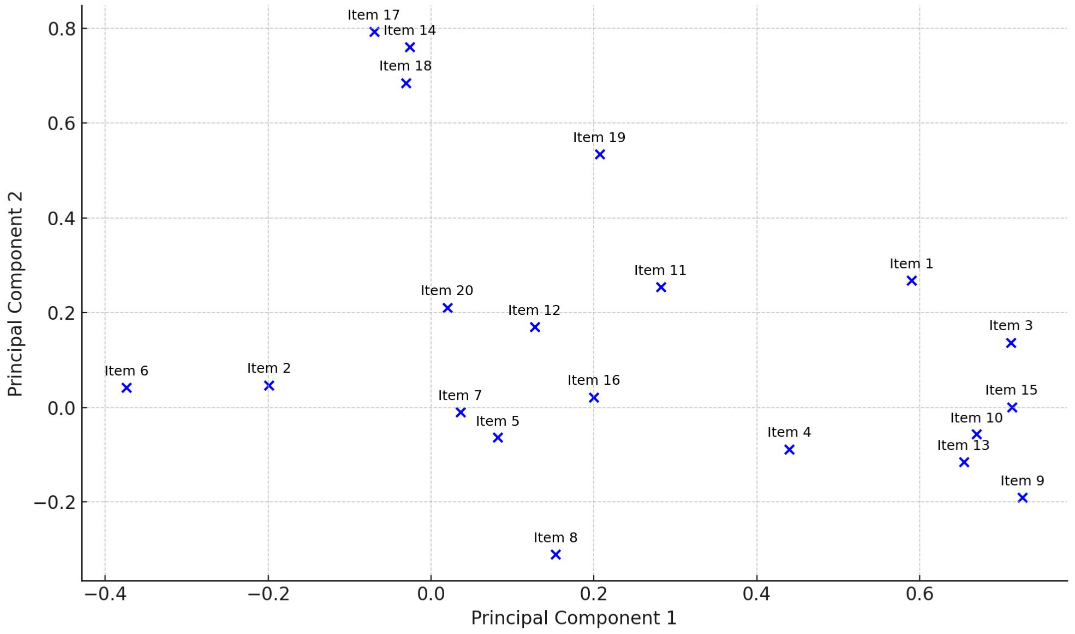

Figure S5. Network plot of Zung SDS from principal component analysis loadings in patients with major depressive disorder.

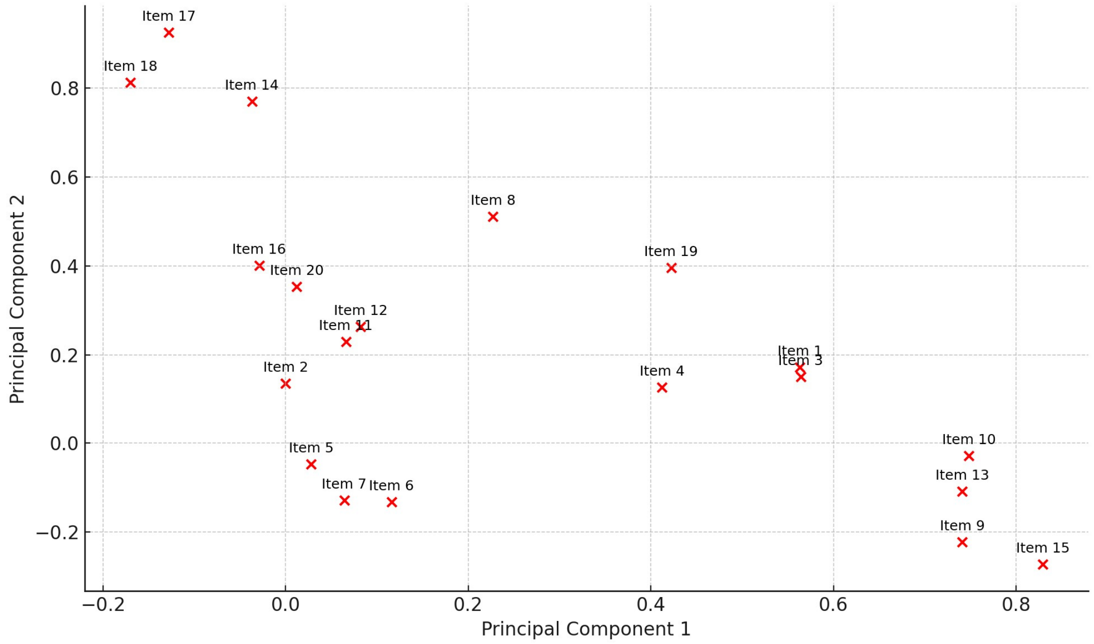

Figure S6. Network plot of Zung SDS from principal component analysis loadings in patients with bipolar disorder.

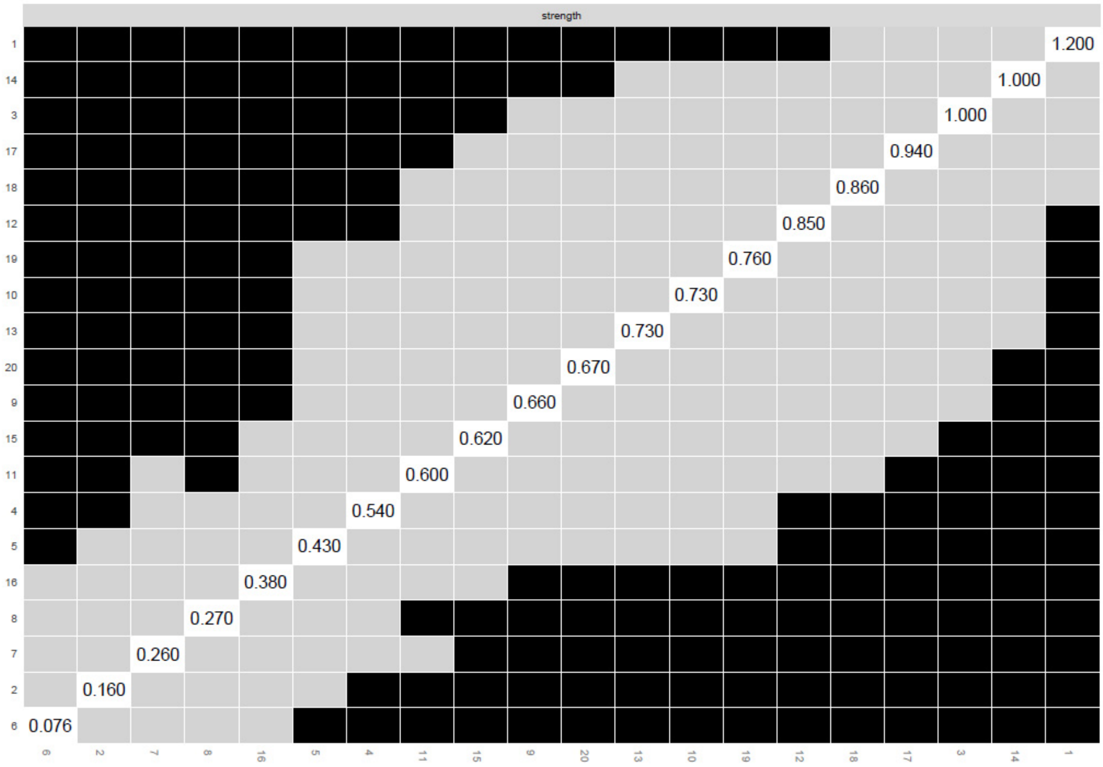

Figure S7. Bootstrapped difference tests between nodes in the Zung SDS 20-symptom-items network among major depressive disorder patients.

Note: Gray boxes indicate that the nodes are not significantly different from each other, while black boxes indicate that the nodes are significantly different (α = 0.05) from each other.

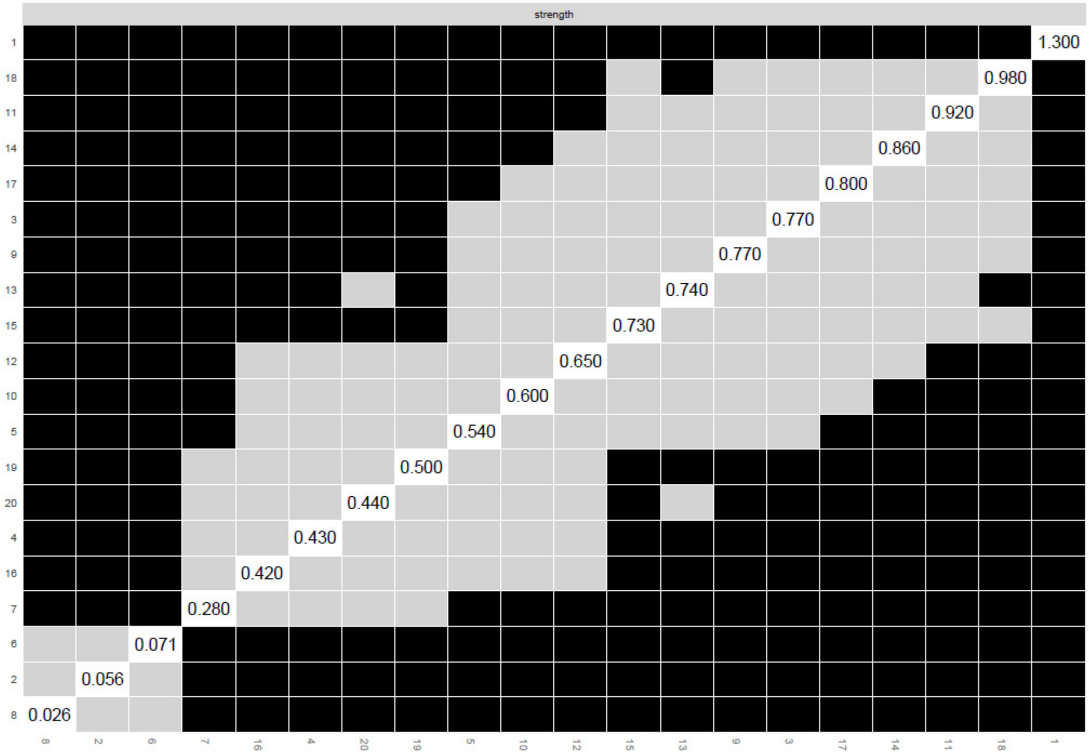

Figure S8. Bootstrapped difference tests between nodes in the Zung SDS 20-symptom-items network among bipolar disorder patients.

Note: Gray boxes indicate that the nodes are not significantly different from each other, while black boxes indicate that the nodes are significantly different (α = 0.05) from each other.

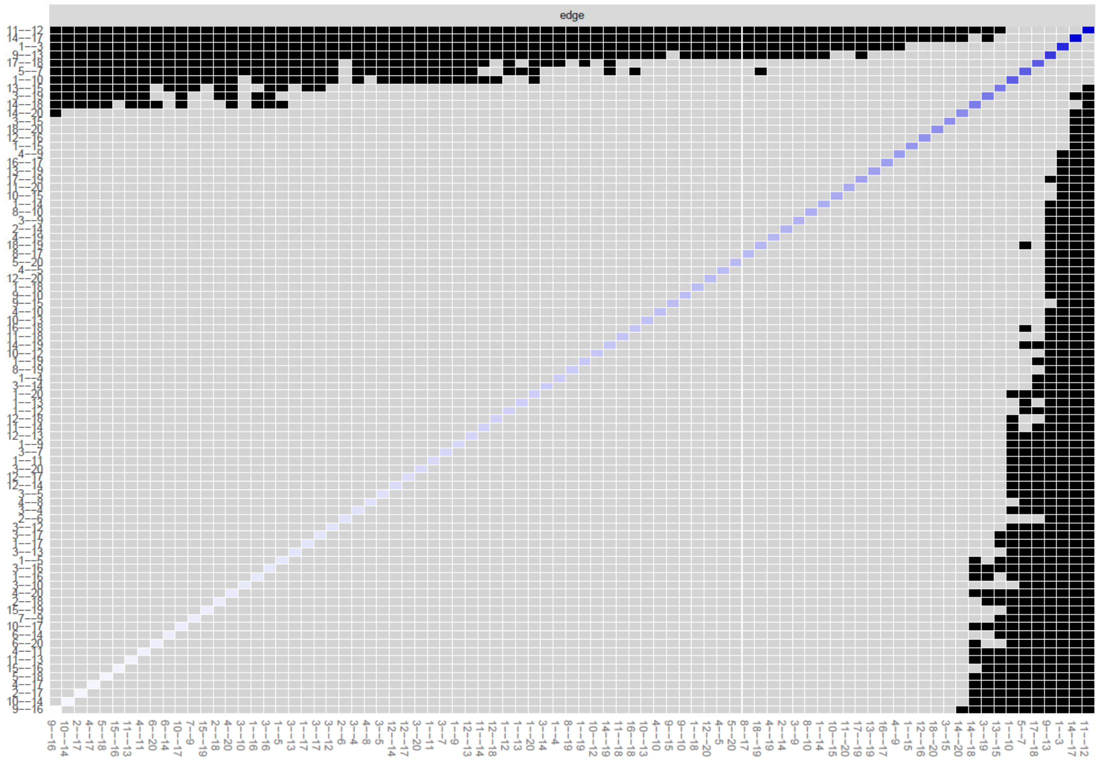

Figure S9. Bootstrapped difference tests between edge-weights that were in the Zung SDS 20-symptom-items network among major depressive disorder patients.

Note: Gray boxes indicate that the nodes are not significantly different from each other, while black boxes indicate that the nodes are significantly different (α = 0.05) from each other.

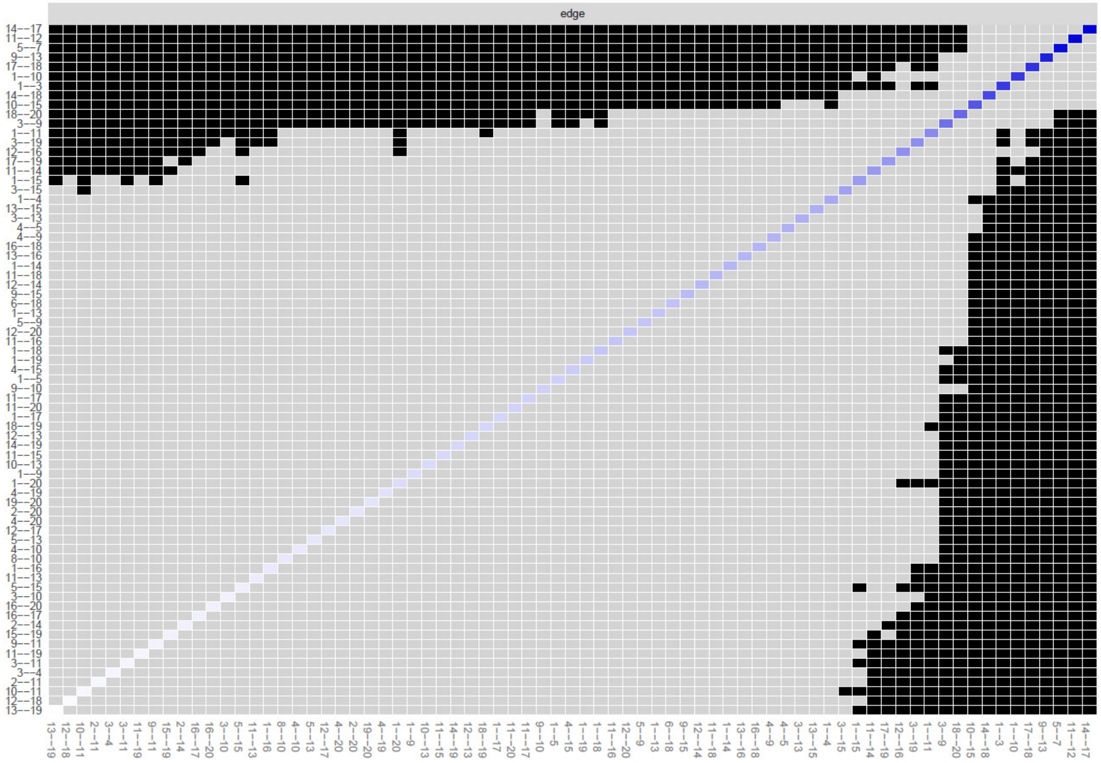

Figure S10. Bootstrapped difference tests between edge-weights that were in the Zung SDS 20-symptom-items network among bipolar disorder patients.

Note: Gray boxes indicate that the nodes are not significantly different from each other, while black boxes indicate that the nodes are significantly different (α = 0.05) from each other.

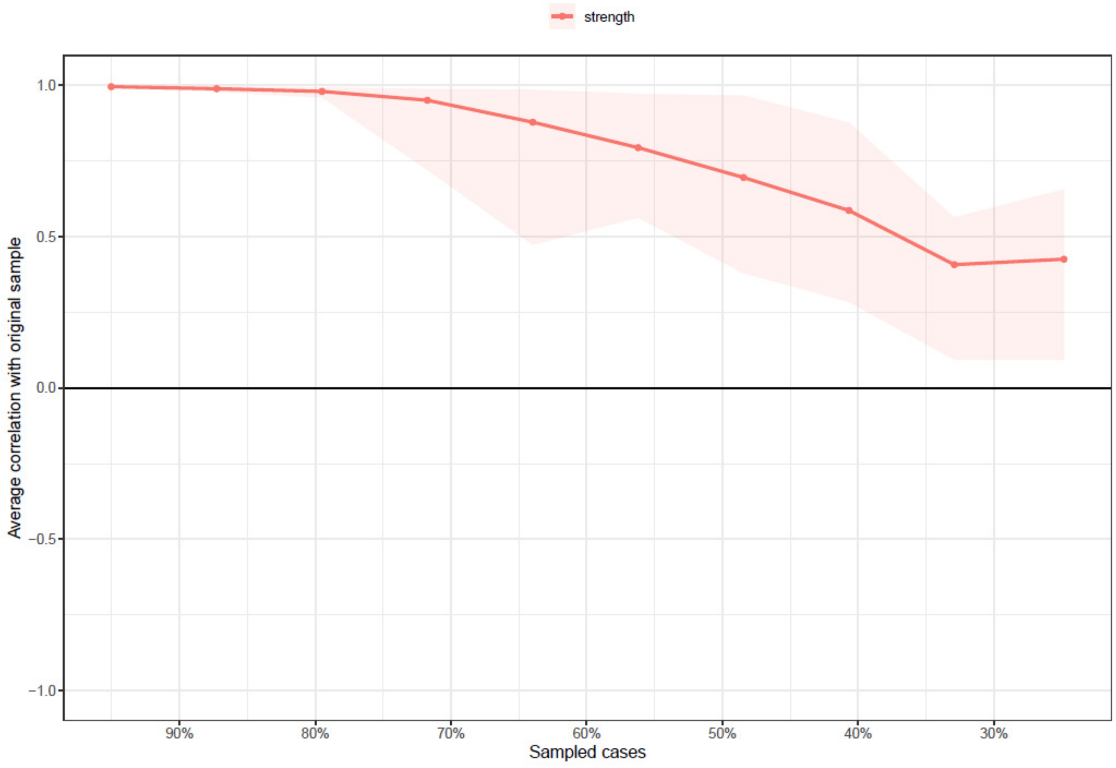

Figure S11. Bootstrapped the strength centrality stability of the Zung SDS 20-symptom-items network among major depressive disorder patients.

Note: The x-axis represents the percentage of cases of the original sample. The y-axis represents the average of correlations between centrality indices in the original sample and sample with case-dropped.

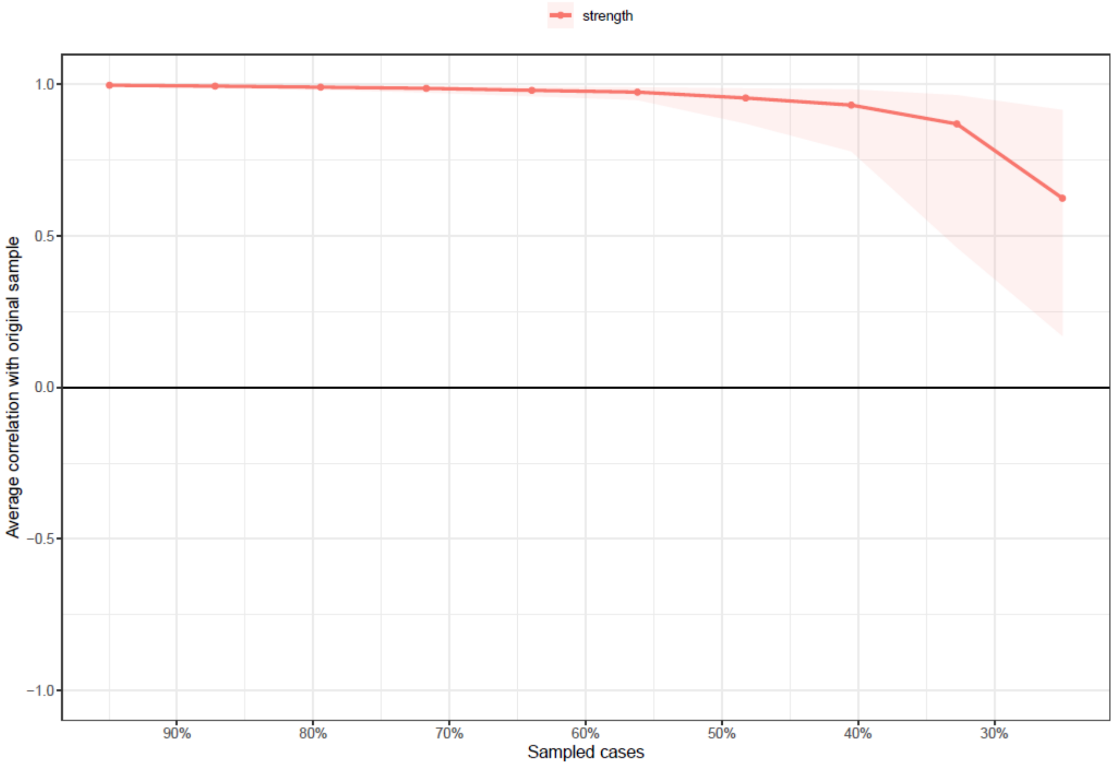

Figure S12. Bootstrapped the strength centrality stability of the Zung SDS 20-symptom-items network among bipolar disorder patients.

Note: The x-axis represents the percentage of cases of the original sample. The y-axis represents the average of correlations between centrality indices in the original sample and sample with case-dropped.
